# Rickettsiosis Caused by *Rickettsia parkeri*, Mexico

**DOI:** 10.3201/eid2802.210454

**Published:** 2022-02

**Authors:** Gaspar Peniche-Lara, Victor Lara-Perera

**Affiliations:** Universidad Autónoma de Yucatán Facultad de Medicina, Merida, Mexico (G. Peniche-Lara);; Hospital General Agustin O’Horan, Secretaria de Salud de Yucatán, Merida (V. Lara-Perera)

**Keywords:** rickettsiosis, Rickettsia parkeri, arboviruses, tick-borne diseases, Mexico, vector-borne infections, viruses

## Abstract

We report a human case of rickettsiosis caused by *Rickettsia parkeri* strain Atlantic Rainforest in Mexico in an adult woman from a small town in the north of Yucatan, Mexico. We confirmed diagnosis using conventional PCR and sequence analysis. Health providers should be aware of clinical manifestations of rickettsioses in this region.

Rickettsiosis caused by *Rickettsia felis*, *R. rickettsii*, and *R. typhi* is commonly reported in Mexico, mostly in the states of Sonora, Sinaloa, and Baja California (*R. rickettsii*) and Yucatan (*R. felis*, *R. rickettsii*, and *R. typhi*) (*1*). Rickettsiosis caused by *R. parkeri* (*R. parketri*) is a recently discovered disease. The first human case was reported in United States in 2004, and the bacterium was subsequently found in South America (*2*–*5*). *R. parkeri* infection is less virulent than infection with *R. rickettsii*, the agent of Rocky Mountain spotted fever*.* Signs and symptoms of *R. parkeri* infection are fever, rash, myalgia, and headache. Presence of eschar lesions at the inoculation site are common (*5*). In Mexico, *R. parkeri* strain Atlantic Rainforest has been identified in *Amblyomma ovale* ticks in Veracruz, *R. parkeri* sensu stricto in *A. maculatum* ticks collected from dogs in Sonora, and *R. parkeri* strain Black Gap in *Dermacentor parumapertus* ticks in Sonora and Chihuahua (*6*). These tick species are well distributed in Mexico; *A. ovale* ticks have been identified in northern, central, and southern areas and *A. maculatum* and *D. parumapertus* ticks in northern and central Mexico (*7*), suggesting that *R. parkeri* could be present in the entire country. We document a human case of *R.* *parkeri* rickettsiosis in the state of Yucatan. 

In January 2020, a 48-year-old woman from the municipality of Dzemul in northeastern Yucatan, where no previous cases of rickettsioses had been reported, went to a local health center because of fever (38°C) persisting for 2 days, myalgia, arthralgia in both legs, and abdominal pain. She had removed 2 ticks from her right upper back 2 days before seeking treatment (Figure). The patient stated that she had not traveled to other states or countries or to other regions within the Yucatan in the previous 2 months. Medics prescribed acyclovir and ibuprofen, but signs and symptoms persisted. The patient was therefore admitted to Hospital General Agustin O’Horan in Merida, the capital city of Yucatan. At the time of admission, the patient had fever (38°C), abdominal pain, diffuse arthralgias without rash, and left axillary lymphadenopathy. No palpable adenomegaly or hepatosplenomegaly were reported. We observed 2 skin lesions (12 × 15 mm and 11 × 16 mm, with erythematous haloes) at the bite site. The patient also reported itching and slight pain at the site of the tick bites (Figure).

**Figure:**
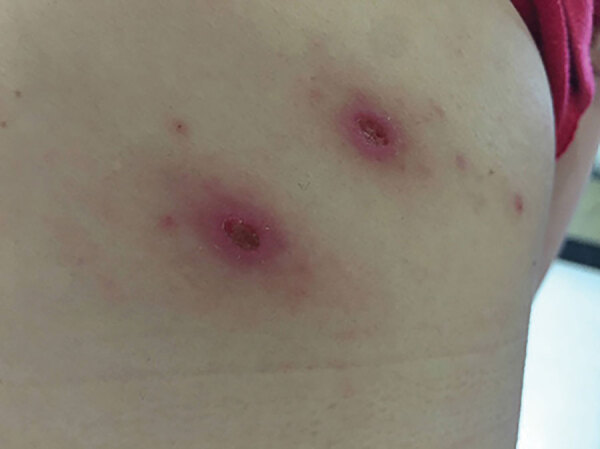
Tick bite sites identified on the right upper back of a 48-year-old woman from the municipality of Dzemul in northeastern Yucatan, Mexico. The woman received a diagnosis of rickettsiosis caused by *Rickettsia parkeri* strain Atlantic Rainforest.

Based on the tick bite and mild infection, the medic suspected rickettsiosis caused by *R. felis*. For conventional PCR diagnosis, we obtained a 3-mL whole blood sample and sent it to our laboratory. In regions where rickettsiosis caused by *R. parkeri* are common, medics usually take samples from eschar lesions (*5*,*8*,*9*). We obtained a skin biopsy from the eschar lesion 2 days after treatment was initiated. However, conventional PCR analysis returned negative results.

We purified DNA from the blood sample following the instructions from a Quick-gDNA MiniPrep Kit (Zymo Research, https://www.zymoresearch.com) and used conventional PCR to amplify 2 gene fragments (*gltA* and *ompB*) for identifying *Rickettsia* spp. Primer sequences and amplification conditions are described by the Latinamerican Guidelines of RIICER for Diagnosis of Tick-Borne Rickettsiosis (*10*). For the *gltA* gene, we amplified a 380-bp fragment and for the *ompB* gene, a 420-bp fragment. We used sterile water as the negative control and *R. typhi* DNA as the positive control. We purified and sequenced products from the PCR. We analyzed DNA sequences using blastn (https://blast.ncbi.nlm.nih.gov/Blast.cgi) and found the amplified products (GenBank accession nos. MW653956 and MW653957) 100% homologous to *R. parkeri* strain Atlantic Rainforest from human cases in Brazil (GenBank accession no. MN027564.1) (*8*) and Colombia (GenBank accession no. MK860201.1) (*5*), as well as to a recently identified *R. parkeri* isolate (GenBank accession no. MK814825.1) from *A. ovale* in Veracruz, Mexico. After diagnosing the *Rickettsia* spp. infection, we treated the patient with doxycycline (100 mg 2×/d for 10 d). After day 2, fever and other symptoms ceased.

## Conclusion

Our study documents a case of human rickettsiosis caused by *R. parkeri* strain Atlantic Rainforest in Yucatan, Mexico. This finding represents the fifth *Rickettsia* species identified as infecting humans in southeastern Mexico, but in a municipality, Dzemul, with no previous *Rickettsia* spp. infections reported among humans or identified in vectors or reservoir hosts. Because this rickettsiosis causes mild to moderate febrile illness with initial symptoms such as fever, headache, muscle pain, nausea, vomiting, rash (*2*), it might masquerade as dengue fever in our region and other areas where dengue is common.

Our report emphasizes the importance of continuing to characterize clinical manifestations of rickettsioses in Mexico. Health providers in this region should include this recently discovered rickettsiosis in differential diagnoses of febrile illnesses.
